# Metal-free aqueous redox capacitor via proton rocking-chair system in an organic-based couple

**DOI:** 10.1038/srep03591

**Published:** 2014-01-07

**Authors:** Takaaki Tomai, Satoshi Mitani, Daiki Komatsu, Yuji Kawaguchi, Itaru Honma

**Affiliations:** 1 Institute of Multidisciplinary Research for Advanced Materials, Tohoku University, Sendai, Miyagi 980-8577, Japan

## Abstract

Safe and inexpensive energy storage devices with long cycle lifetimes and high power and energy densities are mandatory for the development of electrical power grids that connect with renewable energy sources. In this study, we demonstrated metal-free aqueous redox capacitors using couples comprising low-molecular-weight organic compounds. In addition to the electric double layer formation, proton insertion/extraction reactions between a couple consisting of inexpensive quinones/hydroquinones contributed to the energy storage. This energy storage mechanism, in which protons are shuttled back and forth between two electrodes upon charge and discharge, can be regarded as a proton rocking-chair system. The fabricated capacitor showed a large capacity (>20 Wh/kg), even in the applied potential range between 0–1 V, and high power capability (>5 A/g). The support of the organic compounds in nanoporous carbon facilitated the efficient use of the organic compounds with a lifetime of thousands of cycles.

Electrical energy storage systems have numerous applications, including as portable devices, transport vehicles, and stationary energy resources. In particular, interest in electrical energy storage for “smart grids” has grown significantly. Smart grids are promising energy distribution systems that enable a balance between power application and power generation, including renewable energy sources. Renewable energy sources, e.g., solar and wind power, inherently exhibit large and rapid variability, with short-term intermittent spikes and drops. Therefore, for the power grid, a large electrical energy storage system with a high power capability must be installed to smooth the variability^1^,^2^,^3^.

Another important factor in a large stationary application of an electrical energy storage system is cost, which can be subdivided into installation, operation, and maintenance components. The preferred energy storage devices should be composed of inexpensive, easily acquired materials and fabricated through a relatively simple manufacturing process. High durability and reliability coupled with long lifetime and safety are also desirable. Although battery technologies (e.g., lead-acid, sodium-sulfur, redox-flow, and lithium ion batteries) with high energy densities have attracted much attention for grid-scale energy storage, no existing batteries satisfy the above-mentioned requirements. For example, lithium ion batteries, which are the focus of intensive interest as transportation power sources, have unresolved safety issues due to their flammable organic electrolytes. Moreover, battery technologies generally have problems related to rapid charge/discharge.

Capacitor technology can be recognized as the most promising solution for electrical energy storage systems with rapid response^4^,^5^,^6^,^7^,^8^,^9^. A general capacitor can store electrical charge in an electrical double layer (EDL) at the electrode-electrolyte interface. The advantages of capacitors are:

- High power capability
- Excellent lifetimes, exceeding 1000 charge/discharge cycles
- Maintenance-free operation for ~10 years

“Aqueous capacitors,” which work in aqueous electrolytes, have additional advantages:

- High safety due to non-flammable aqueous electrolyte
- Low manufacturing cost (fabrication under ambient conditions without requiring an oxygen- or humidity-free environment)

To maximize the interface area in the electrode for the enhancement of energy density, high-surface-area electrode materials, e.g., activated carbon, are employed. However, the energy density of aqueous capacitors based only on EDL formation is not high (<5 Wh/kg) due to a limiting operating voltage of about 1.23 V at which water decomposes. Under the developed technologies, their combined use with battery systems having high energy densities is necessary for energy storage systems in the power grid. If the energy density of the capacitor exceeds at least that of lead acid batteries (20–40 Wh/kg), the current combined energy storage system will be replaced by a capacitor-only device.

To enhance the energy density of energy storage devices, electrodes containing active redox-reactive materials, e.g., metal oxides, have been investigated. However, large-scale installations using current redox materials consisting of rare metals, e.g., LiCoO_2_ in lithium ion batteries^10^,^11^ or RuO_2_ and MnO_2_ in electrochemical capacitors^5^,^9^,^12^,^13^, would leave large ecological footprints and face resource restrictions. Against this background, inexpensive organic compounds have recently attracted much attention as redox-active materials for secondary batteries^14^,^15^,^16^ and electrochemical capacitors^17^,^18^,^19^,^20^. Unlike the redox-active materials based on metal oxides, organic compounds are free from resource restrictions and potentially environmentally benign. Moreover, many redox-active organic materials possess large ion storage capacities beyond those of conventional redox-active metal oxides, primarily owing to the multielectron reactions in a low-molecular-weight moiety.

Among the redox-active organic materials, the ketone group is known to show fast redox reactions with protons in aqueous capacitors^21^. Quinones can facilitate two-proton reactions in a molecule. Reports have shown that both the capacitance and energy density were enhanced by the redox reactions of quinones loaded in activated carbon via impregnation^17^,^20^, grafting^18^,^19^, or dissolution in the electrolyte^22^,^23^. However, most such redox capacitors consisted of at least one unmodified or unloaded carbon electrode, and redox capacitors configured from two different electrodes employing a couple based on two organic compounds having different reaction potentials have rarely been investigated, despite their promise for further increases in energy density.

In this work, we present a metal-free redox capacitor using a couple comprising low-molecular-weight organic compounds in an aqueous electrolyte. In the capacitor, shown schematically in Fig. 1, each electrode consisted of a different organic compound impregnated in nanoporous activated carbon. The quinone/hydroquinone couple employed consisted of anthraquinone (AQ) and tetrachlorohydroquinone (TCHQ) as the active materials with different redox potentials for proton insertion/extraction reactions. Protons in the aqueous electrolyte played the roles of charge carriers. This energy storage mechanism, in which protons are shuttled back and forth between two electrodes upon charge and discharge, can be regarded as a proton rocking-chair system. The nanoporous carbon facilitated the high utilization rate of the redox reactions of the organic compounds simply impregnated in the carbon at high loading. The manufactured capacitor exhibited promising performance in terms of high energy density, high power density, and long cycle lifetime.

## Results

We conducted the galvanostatic charge/discharge cycling of the redox capacitor fabricated with an AQ and TCHQ couple in a three-electrode configuration. The electrodes consisted of the organic compound (27 wt%), the nanoporous carbon (63 wt%), and polytetrafluoroethylene (PTFE, 10 wt%). PTFE was employed as a binder to prepare a paste of the organic compound-carbon composite. Both electrode weights were identical. The positive and negative electrodes contained TCHQ and AQ impregnated in the nanoporous carbon, respectively. During the charge process, AQ acted as a proton acceptor and was transformed into anthrahydroquinone, whereas the proton-donor TCHQ was transformed to tetrachlorobenzoquinone. During the discharge process, the roles were reversed, and both materials were restored to their initial states.

**Figure 2:**
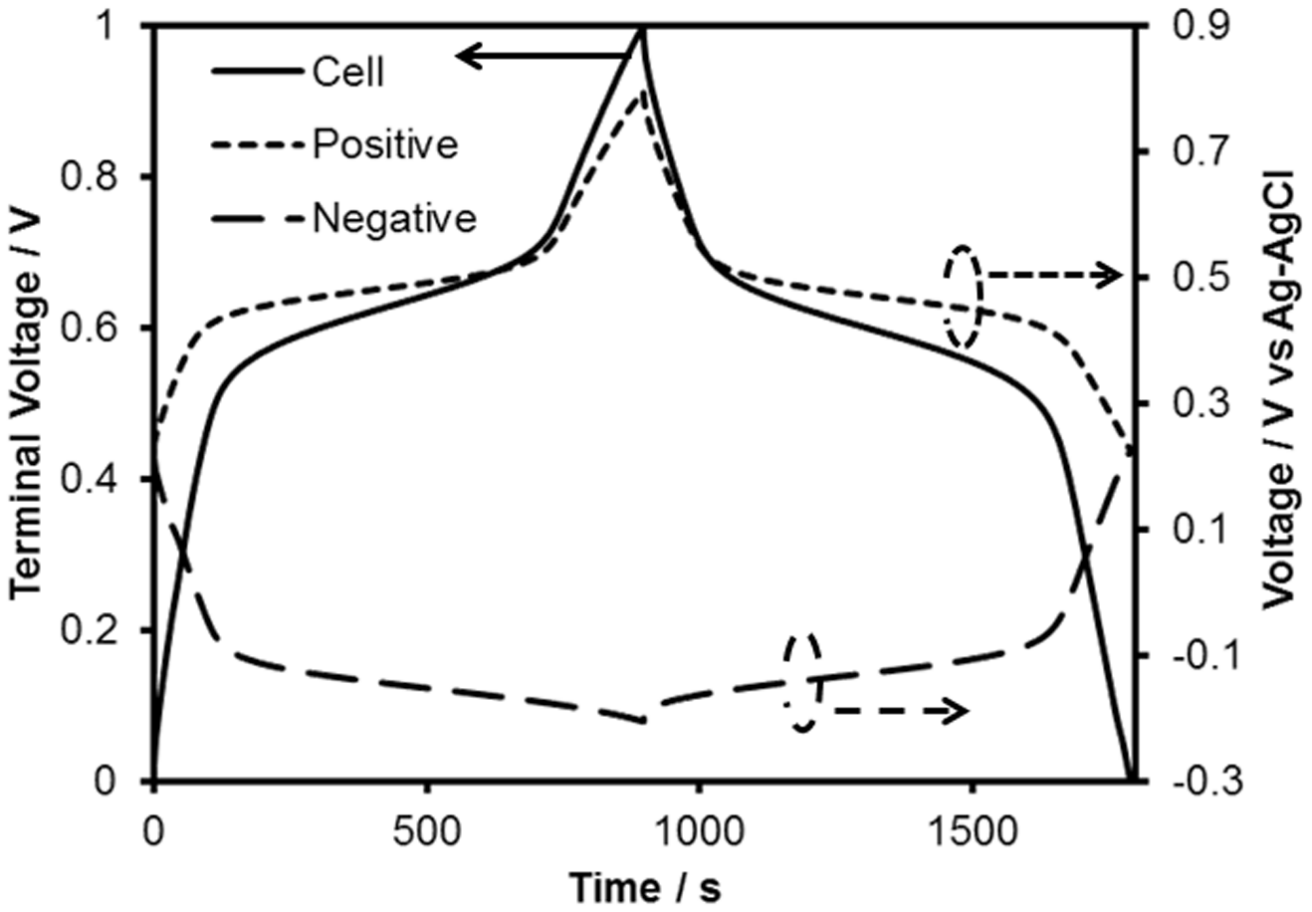
Galvanostatic cycle of the redox capacitor and evolution of the potential corresponding to the positive (TCHQ) and negative (AQ) electrodes in 0.5 M H_2_SO_4_ aqueous solution at 0.28 A/g.

Figure 2 shows the typical galvanostatic cycle and the evolution of the potential corresponding to the positive and negative electrodes in 0.5 M H_2_SO_4_ aqueous solution. The plateau potentials of the positive and negative electrodes corresponded to the redox peaks of TCHQ (0.50 V vs. Ag/AgCl) and AQ (−0.16 V vs. Ag/AgCl) observed in the cyclic voltammograms (see Supplementary Fig. S1 online), and the plateau potential in the galvanostatic cycle (at ~0.65 V) corresponded to their difference. These behaviors indicated that the proton-extraction/insertion reaction of TCHQ occurred at the positive electrode simultaneously with the proton-insertion/extraction reaction of AQ at the negative electrode, and the energy storage of this capacitor relies on a proton rocking-chair mechanism. The rechargeable energy density of this redox capacitor based on the total weight of two electrodes was approximately 20.3 Wh/kg at a current density of 0.28 A/g. In contrast, the rechargeable energy of an EDL capacitor, which had a symmetric configuration and consisted of nanoporous carbon without the organic compounds, was approximately 6.7 Wh/kg at the similar current density (0.34 A/g) (see Supplementary Fig. S2 online). Thus, the energy density of the capacitor increased three-fold via the impregnation of the organic compounds.

The calculated energy density of the redox capacitor includes both the contribution from the EDL capacity in addition to that from the redox reactions of the organic compounds. To focus on the capacity of electrical charge derived only from the organic compounds, we estimated the EDL capacity by extrapolating the galvanostatic discharge profile in the range from 0 to 0.4 V, where the redox reactions did not take place. The near-linear profile in this range should be derived from the EDL of the nanoporous carbon support. The capacities of electrical charge derived from the EDL and the redox reactions can be estimated to be 34.8 mAh/g_-carbon_ based on the carbon weight in single electrode and 186 mAh/g_-(AQ or TCHQ)_ based on the organic compound weight in single electrode, respectively. The calculation detail is in the online Supplementary Information (Fig. S3). The capacity derived from the EDL in the redox capacitor was nearly the same as that observed for the pure EDL capacitor (32.6 mAh/g_-carbon_). The ideal proton storage capacities of AQ and TCHQ with two-proton redox reactions were 257 mAh/g_-AQ_ and 216 mAh/g_-TCHQ_, respectively. Because the weights of the organic compounds were identical, the capacity derived from the redox reactions should be limited by the TCHQ capacity. Therefore, the utilization rate of the organic compounds in our redox capacitor can be regarded to be approximately 86% based on the weight of TCHQ.

**Figure 3:**
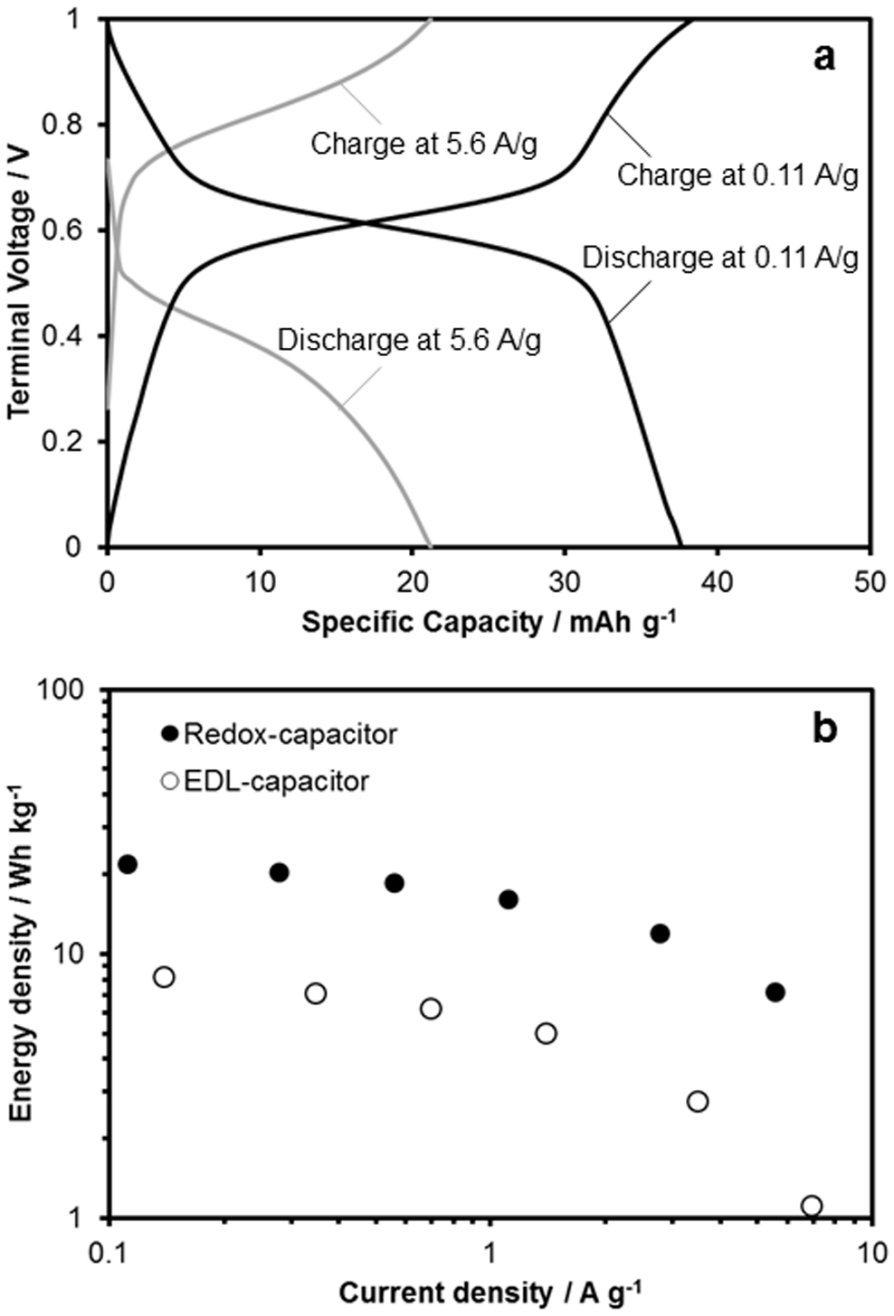
(a) Galvanostatic cycles of the redox capacitor (TCHQ-AQ) in 0.5 M H_2_SO_4_ aqueous solution at 0.11 and 5.6 A/g, and (b) the rechargeable energy density of the redox capacitor (TCHQ-AQ) and the pure EDL capacitor (consisting of the nanoporous carbon) as a function of current density.

We examined the rapid response capability of this redox capacitor. As mentioned, a high power capability is essential to level the variability from renewable energy sources. Aqueous capacitors are potentially advantageous for rapid charge/discharge owing to the high ionic conductivity of the aqueous electrolyte. Figure 3a shows the galvanostatic cycles at 0.11 and 5.6 A/g. Both displayed similar profiles, consisting of plateau or plateau-like regions derived from the redox reactions of the organic compounds, and linear regions derived from EDL formation in the charge and discharge conditions. These profiles mean the proton insertion/extraction reaction also occurs even at high current density, and the proton rocking-chair mechanism using the organic-based couple is effective for rapid response. It should be noted that the plateau potentials at the charge and discharge conditions, which were nearly equal at low current density, moved apart from each other as the current density increased. This separation (polarization) is considered to be derived both from the electrolyte resistance and the overpotential of the redox reaction of the organic compounds, and deteriorates the rechargeable energy density of our redox capacitor. Figure 3b shows the rechargeable energy density of the redox capacitor and a pure EDL capacitor consisting of the nanoporous carbon at various current densities. As the current density increased, the rechargeable energy density decreased (21.8 Wh/kg at 0.11 A/g and 7.15 Wh/kg at 5.6 A/g). However, the capability of the redox capacitor for high current density was similar to that of the pure EDL capacitor. The fast redox reactions of quinones should contribute to this outstanding capability, which is comparable to that of EDL capacitors.

## Discussion

Support of the organic compounds in the nanoporous carbon should play a crucial role for the effective use of the capacity of organic compounds. Because of the low- or non-conductivity of AQ and TCHQ, their crystalline bulk could not be utilized for electrochemical redox reactions without a carbon support. However, as indicated above, the organic compounds held in nanoporous carbon contributed to the energy storage. X-Ray diffraction (XRD) and^1^H-NMR studies revealed that the most of the organic compounds were held on the surface of the nanoporous carbon with less-crystalline or nanocrystalline structures (see Supplementary Figs. S4–S7 online). Because the proportion of the organic molecules contacting the carbon surface to the total of the loaded organic molecules increases by downsizing the supporting pores, the interaction between the sp^2^ carbon surfaces and the aromatic rings will become apparent and stabilize the absorbed organic compounds in the nanometer-scaled pores. The organic compounds held in carbon materials with less-crystalline or nanocrystalline structures showed high utilization rate (see Supplementary Figs. S8 and S9 online). It is highly anticipated that such adsorption states of the organic compounds on a carbon material with electrical conductivity contribute to the redox reactions with a high utilization rate.

We also evaluated the dependence of the cycle lifetime on the kinds of carbon materials for a single electrode system (see Supplementary Fig. S8). In the measurements, AQ impregnated in the carbon materials was employed in the working electrode, where the excess amount of carbon material was employed in the counter electrode. For a carbon material with a larger averaged pore size (*ϕ*11 nm), the retention rate of capacitance for the working electrode after 1000 charge/discharge cycles at 0.56 A/g was 41%. For the nanoporous carbon material (averaged pore size: *ϕ*2.2 nm) employed in this study, the retention rate after 1000 cycles was 82%. From this comparison, we found that the support of organic compounds in the nanoporous carbon also contributed to the cycle lifetime enhancement of the redox capacitor.

**Figure 4:**
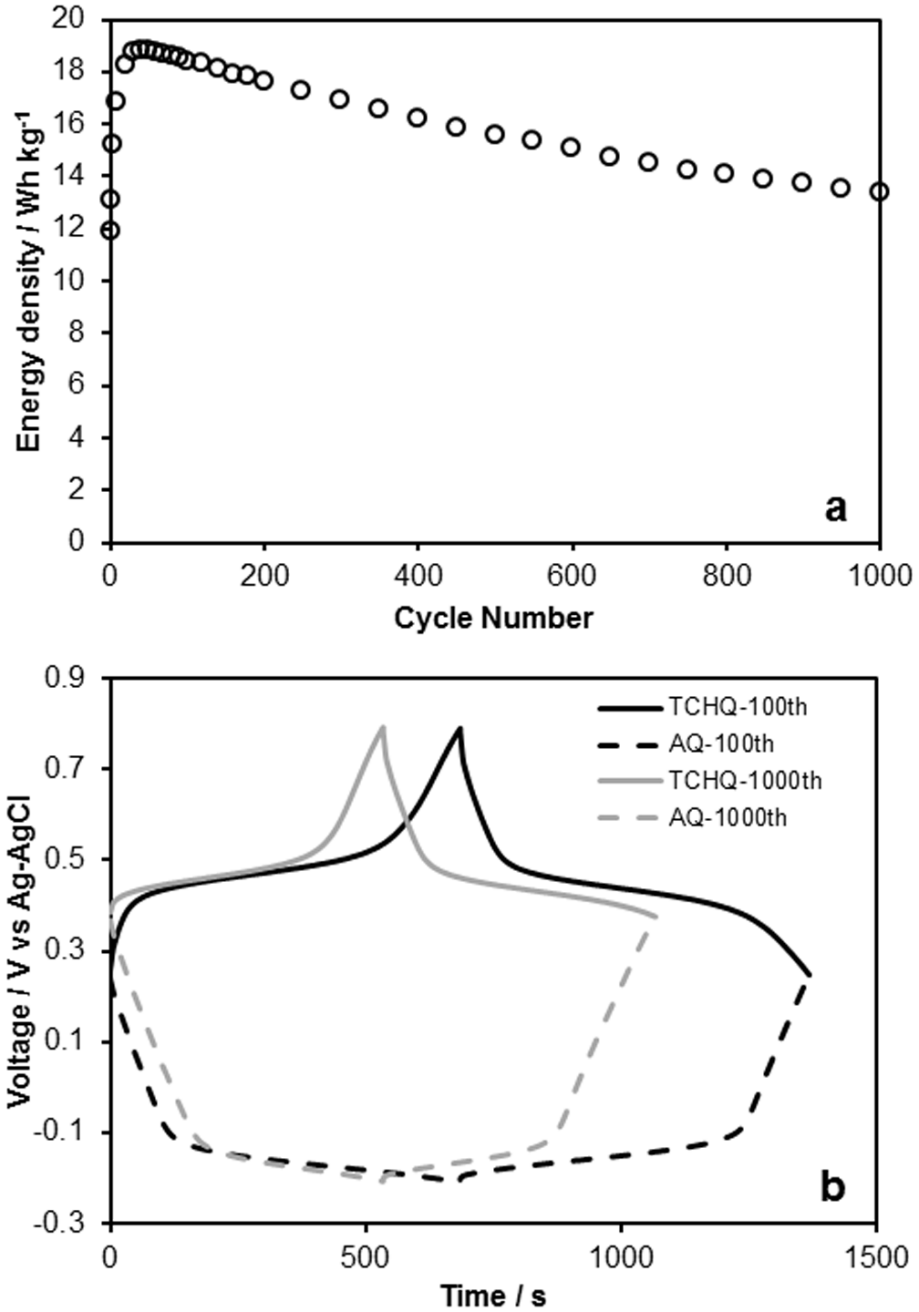
(a) Rechargeable energy density of the redox capacitor (TCHQ-AQ) in 0.5 M H_2_SO_4_ aqueous solution at 0.28 A/g as a function of cycle time, and (b) the evolution profiles of the potential corresponding to the positive (TCHQ) and negative (AQ) electrodes at the 100^th^ and 1000^th^ cycles.

Durability becomes a critical issue when using organic compounds as the redox-active materials, because of their solubility. Generally, as the molecular weight decreases, the solubility of quinones in aqueous solution generally increases. For example, the solubilities of benzoquinone and AQ in water at 25°C are approximately 10 g/l and 1.4 mg/l, respectively. Moreover, hydroquinones, which forms by proton insertion reaction with quinones, are more soluble than quinones (the solubility of hydroquinone in water at 25°C are 80 g/l). Therefore, the dissolution of organic compounds during charge/discharge degrades the cycle performance of the aqueous capacitor that uses such compounds. It was supposed that the support by carbon in the nanometer-scaled pores would be effective against dissolution. It is known that the nanoporous carbon employed in this study has a complicated branching pore structure^24^. Support inside this structure would prevent organic compounds from dissolving out of the carbon matrix, and enable their reversible redox reactions with long cycle lifetimes. Figure 4a shows the rechargeable energy density of the redox capacitor device as a function of cycle time. The coulombic efficiency was above 99% for almost all charge-discharge cycle. At a current density of 0.28 A/g, the energy density exhibited an initial growth until about the 100^th^ cycle, and then gradually decreased. At present, the delay time that it takes for the redox capacitor to show its maximal energy density has not been fully controlled, and remains a challenge. After 1000 cycles, the retention rate based on the maximum energy density was approximately 70%.

Finally, we suggest a strategy for further enhancement of cycle lifetime. Figure 4b shows the evolution of the potential corresponding to the positive and negative electrodes. The final potential after the discharge process completed at the 1000^th^ cycle, 0.38 V vs. Ag/AgCl, was much higher than that at the 100^th^ cycle, 0.25 V vs. Ag/AgCl. This increase in the final potential (0.13 V) implies that the decrease in the rechargeable energy density after many cycles results from the degradation of the negative electrode, because the evolution of the potential for the negative electrode caused by its shortened plateau region interrupts the full-capacity charge/discharge of the positive electrode. It was conjectured that the degradation of the negative electrode could be attributed to the dissolution of AQ into the aqueous electrolyte as anthrahydroquinone. Therefore, instead of AQ, we employed 1,5-dichloroanthraquinone (DCAQ) as the redox-active material in the negative electrode. The attachment of hydrophobic functional groups was effective in minimizing dissolution of the organic compounds into the aqueous solution. For example, the solubility of TCHQ, which has 4 hydrophobic chloro groups, in water at 25°C is quite low, 76 mg/l, compared with that of hydroquinone (80 g/l).

**Figure 5:**
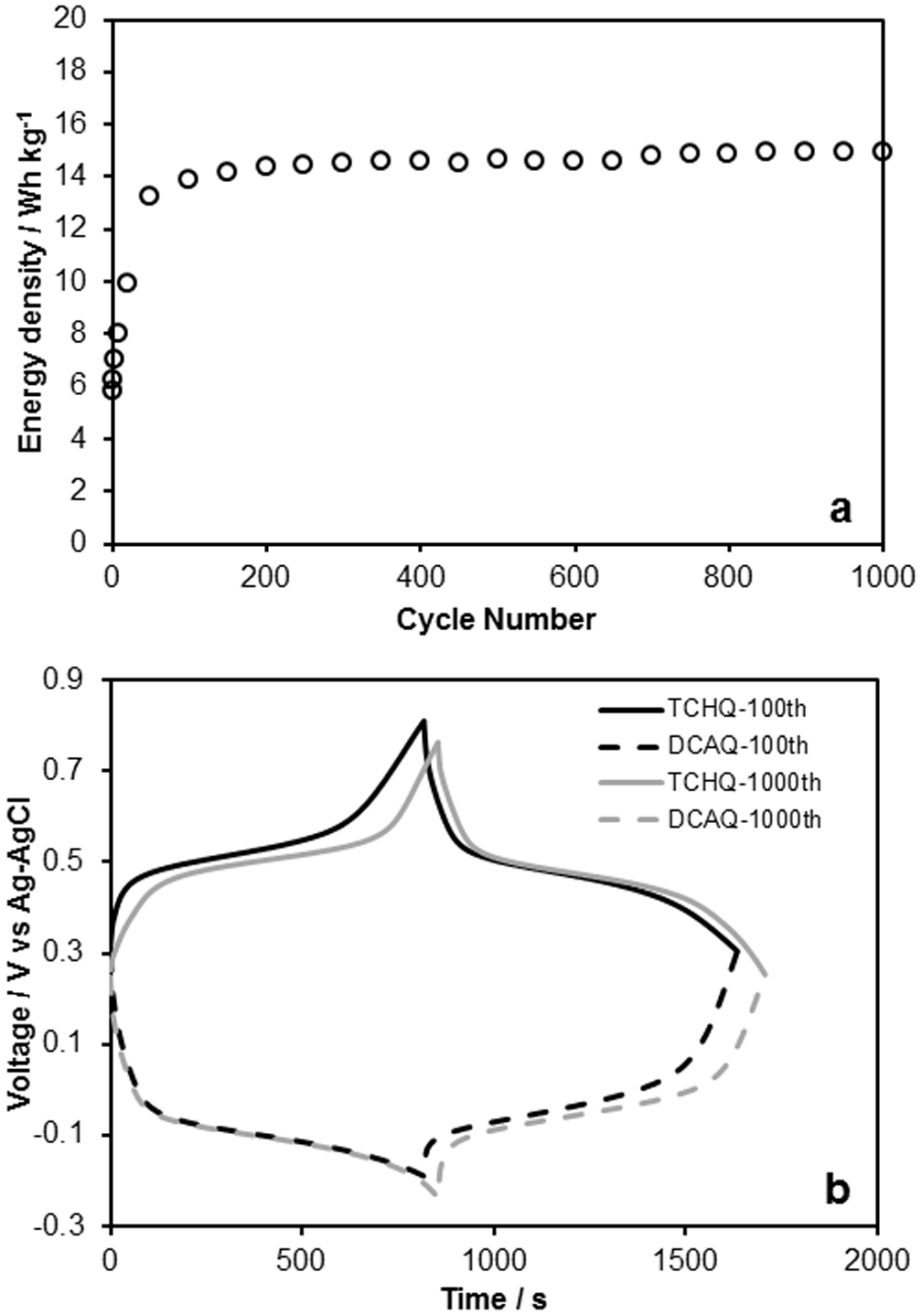
(a) Rechargeable energy density of the redox capacitor (TCHQ-DCAQ) in 0.5 M H_2_SO_4_ aqueous solution at 0.26 A/g as a function of cycle time, and (b) the evolution profiles of the potential corresponding to the positive (TCHQ) and negative (DCAQ) electrodes at the 100^th^ and 1000^th^ cycles.

Figure 5a shows the rechargeable energy density of the redox capacitor using the DCAQ and TCHQ couple as a function of cycle time at 0.26 A/g. The coulombic efficiency was above 99% for almost all charge-discharge cycle. Similar to the redox capacitor that used the AQ and TCHQ couple, the energy density reached its maximum around at the 100^th^ cycle. On the other hand, compared with the case of AQ/TCHQ, the cycle performance was enhanced by the attachment of the hydrophobic chloro groups, and the degradation of the rechargeable energy density was not observable after 1000 cycles. In addition, the cycle performance of the redox capacitor (TCHQ-DCAQ) at 2.6 A/g, the degradation of the rechargeable energy density was not observable after 10000 cycles (see Supplementary Fig. S10). These results suggest that the attachment of hydrophobic functional groups was effective as a strategy for the further enhancement of cycle lifetime. Figure 5b shows a typical galvanostatic cycle and the evolution of the potential corresponding to the positive and negative electrodes in the redox capacitor. The potentials of the redox reactions with protons were −0.05 V (vs. Ag/AgCl) for DCAQ and 0.50 V (vs. Ag/AgCl) for TCHQ. Therefore, the potential plateaus around 0.55 V corresponded to the difference of the redox reaction potential between DCAQ and TCHQ, and it was confirmed that the energy storage of this capacitor relies on the redox reactions of DCAQ and TCHQ. Compared with the AQ and TCHQ couple, the decrease in the energy density resulted from the decrease in the potential difference. However, in this case, the decrease in the final potential after the discharge process was not observed. This indicated that the suppressed solubility of DCAQ by the attachment of hydrophobic functional group contributed to avoid the degradation of the negative electrode and the interruption of the full-capacity charge/discharge of the positive electrode.

In summary, we demonstrated an aqueous redox capacitor using a couple comprising organic compounds that enabled a proton rocking-chair-type energy storage. Fundamentally unlike conventional batteries and supercapacitors, the employed electrodes, which consisted only of light elements (hydrogen, carbon, oxygen, and chlorine), can be installed with low material and manufacturing costs. By employing nanoporous carbon, a redox capacitor with a long cycle lifetime and a high energy density, derived from the high utilization rate of the redox reaction of the organic compounds, was achieved. Moreover, this capacitor can respond to rapid variability. These features are promising for large stationary applications in electrical energy storage systems for the energy grid.

Furthermore, we proposed a strategy to further enhance cycle lifetime by controlling quinone/hydroquinone solubility. In addition to hydrophobicity, the required properties of the organic compounds in the capacitor were low molecular weights, ability to participate in fast redox reactions for high energy density, and high power capability. The coupling of different organic compounds having different redox potentials in the limiting operating voltage of an aqueous solution was also important. Further improvements via the choice of the best organic-based couple and the optimized loading of the organic compounds on carbon will accelerate the replacement of current electrical energy storage systems for grid use.

## Methods

### Preparation of organic compounds supported by carbon materials

Nanoporous activated carbon (Maxsorb® MSC-30, Kansai Coke and Chemicals Co., Ltd.) was employed as the support for the organic compounds. N_2_ adsorption/desorption behaviors obtained by an automatic adsorption apparatus (BELSORP-18, BEL Japan, Inc.) revealed that the nanoporous activated carbon had a BET surface area of 3070 m^2^/g and a pore volume of 1.70 cm^3^/g. In addition, BJH analysis indicated that the number of pores larger than 10 nm was negligible.

To prepare the organic compound-carbon composites, the as-received organic compound (AQ, TCHQ, or DCAQ (Tokyo Chemical Industry Co., Ltd.)) was dissolved or dispersed in acetone with the carbon material by sonication. A detailed discussion of the selection of these compounds is included in the online Supplementary Information (Table S1 and Figs. S11 and S12). The weight ratio of the carbon material to the organic material was fixed at 7:3. By evaporating the acetone at 70°C, the organic compound was supported in the carbon material.

### Electrochemical measurement setup

The electrodes were prepared in the form of pressed pellets (*ϕ*7 mm) using a mixture of organic compound-carbon composite and polytetrafluoroethylene (PTFE) as a binder. The weight ratio of the composite to PTFE was fixed at 9:1, and the total weight of an electrode is fixed to be approximately 4 mg. The electrodes were attached onto Au mesh current collectors. Aqueous H_2_SO_4_ solution (0.5 M) and a Ag/AgCl electrode were employed as the electrolyte and the reference electrode, respectively. Electrodes are inserted into 70 ml electrolyte in glass tube. After degassing the electrodes and electrolyte under vacuum until the open circuit potential (OCV) became stable, we initiated measurements using a potentiostat/galvanostat (VMP3, Bio-Logic) under ambient conditions. The applied potential range between the positive and negative electrodes was 0–1.0 V. To remove the oxygen dissolved in the aqueous electrolyte, N_2_ gas was bubbled through the electrolyte throughout the charge-discharge measurements. The current density calculations were based on one electrode weight. The energy density calculations were based on the total weight of both electrodes.

## Author Contributions

D.K., M.S. and Y.K. performed and analyzed electrochemical and physical measurements. T.T., M.S. and I.H. designed and analyzed electrochemical and physical characterizations. T.T. wrote this paper. All authors reviewed this manuscript.

## Supplementary Material

Supplementary Information Supplementary Information

## Figures and Tables

**Figure 1:**
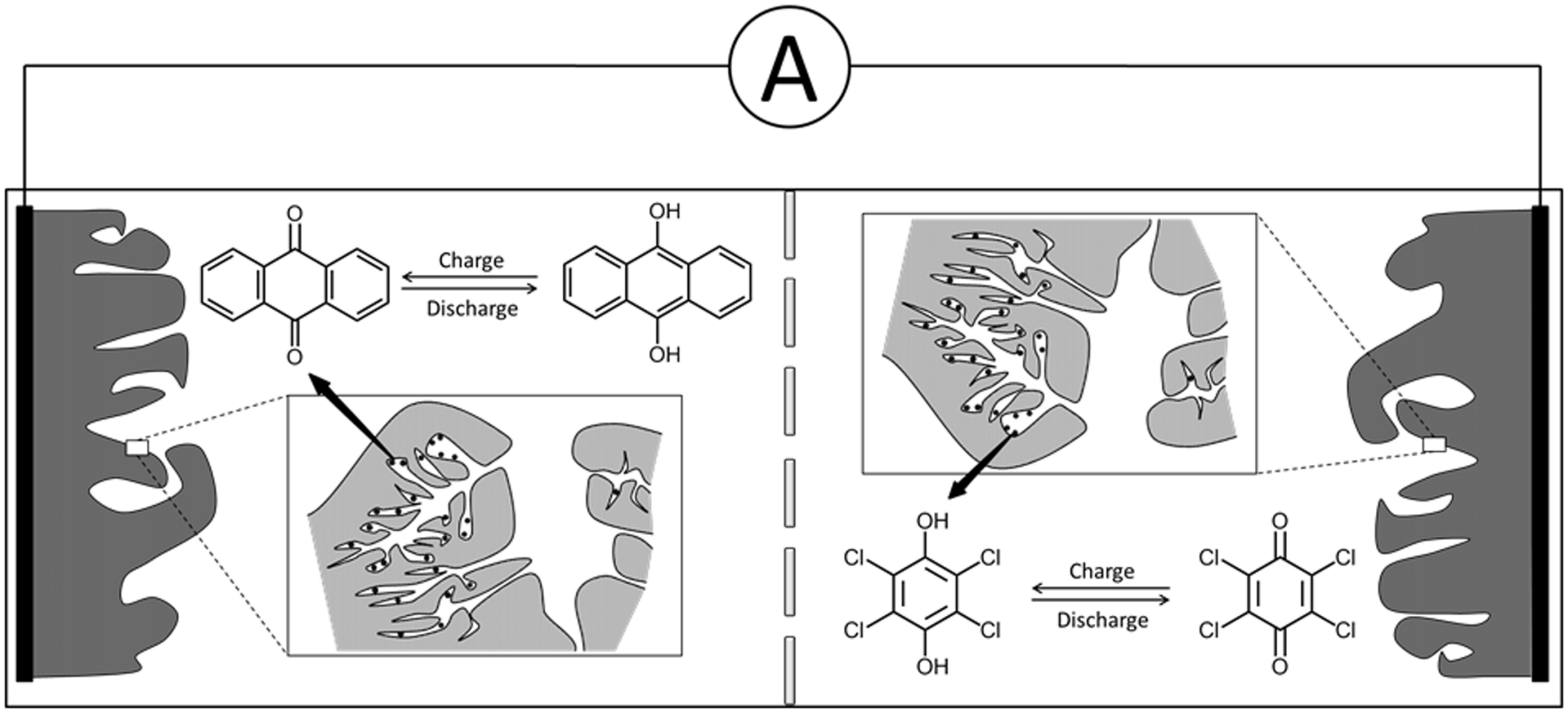
Schematic of the metal-free redox capacitor using a couple based on low-molecular-weight organic compounds in an aqueous electrolyte.
