# Supplementary material for: Metal-free aqueous redox capacitor via proton rocking-chair system in an organic-based couple
Source: Sci Rep. 2014 Jan 7;4:3591. doi: 10.1038/srep03591 (PMC3882745; doi:10.1038/srep03591)
Supplement: Supplementary Information [file srep03591-s1.pdf]

Supplementary Information

Metal-free aqueous redox capacitor via proton rocking-chair system in an organic-based couple

Takaaki Tomai, Satoshi Mitani, Daiki Komatsu, Yuji Kawaguchi, and Itaru Honma**

Institute of Multidisciplinary Research for Advanced Materials, Tohoku University,

Sendai, Miyagi 980-8577, Japan

tomai@tagen.tohoku.ac.jp, i.honma@tagen.tohoku.ac.jp

1. Cyclic voltammetry measurements for redox potentials of TCHQ and AQ

In the electrochemical measurements for the single electrode characteristics, a pressed paste (consisting of nanoporous activated carbon (Maxsorb®), carbon black (Ketjenblack®), and PTFE) on the Pt current collector was employed as a counter electrode. The weight ratio of Maxsorb, Ketjenblack, and PTFE was fixed at 8:1:1, and the total weight was greater than ten times that of the working electrode to suppress the potential change of the counter electrode during charge/discharge processes. Aqueous H_2SO_4 solution (0.5 M) was employed as the electrolyte. A Ag/AgCl electrode was employed as the reference electrode. After degassing the electrodes and electrolyte under vacuum until the open circuit potential (OCV) became stable, we initiated cyclic voltammetry measurements under ambient conditions without N_2 bubbling. The scan rate was 1 mV/s.

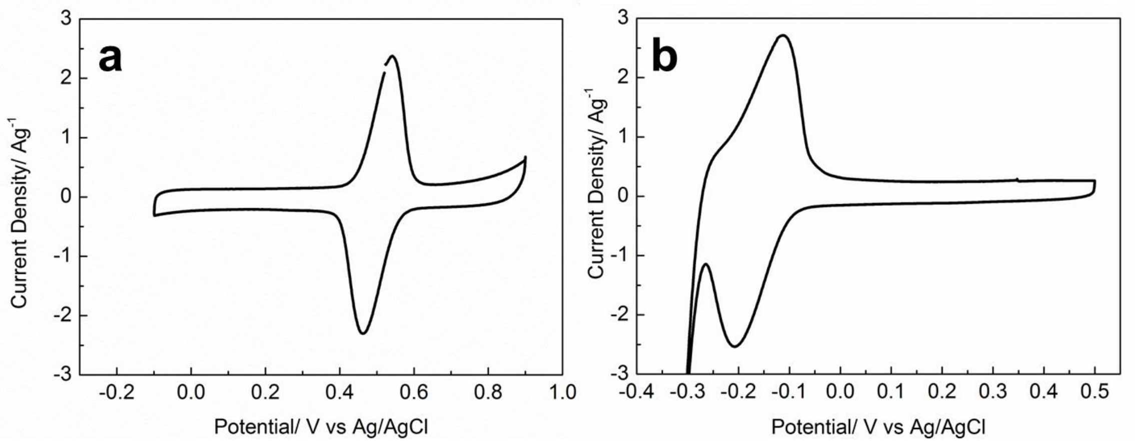

Supplementary Fig. S1 Cyclic voltammograms for (**a**) positive and (**b**) negative electrodes containing TCHQ and AQ, respectively.

Cyclic voltammograms for the positive electrode containing TCHQ and the negative electrode containing AQ are shown in Supplementary Fig. S1. In Supplementary Fig. S1**a**, the anodic and cathodic peak potentials were 0.54 and 0.46 V (vs. Ag/AgCl), respectively. Therefore, the redox potential of the proton insertion/extraction reaction for TCHQ was estimated as the averaged value of the potentials, 0.50 V.

In contrast, the anodic and cathodic peak potentials in Supplementary Fig. S1**b** were −0.11 and −0.21 V (vs. Ag/AgCl), respectively. Thus, the redox potential of the proton insertion/extraction reaction for AQ was estimated to be −0.16 V.

2. Pure EDL capacitor

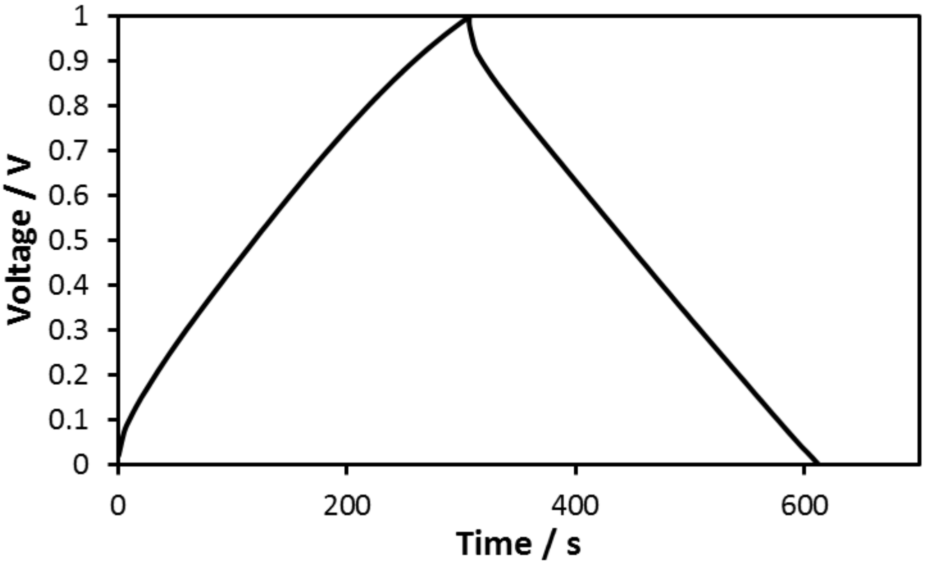

Supplementary Fig. S2 Galvanostatic cycle of EDL capacitor in 0.5 M H_2SO_4 aqueous solution at 0.34 A/g.

For comparison, we fabricated pure EDL capacitor which had a symmetric configuration and consisted of nanoporous carbon without the organic compounds. The galvanostatic cycle is shown in Supplementary Fig. S2. The weight of nanoporous carbon in one electrode is approximately 4.1 mg, and the total weight of both electrodes including PTFE binder is 9.2 mg. The charge and discharge current was fixed to be 1.582 mA. Therefore, the current density is 0.34 A/g based on the weight of single electrode (4.6 mg). The charge and discharge in the range from 0 to 1.0 V has been completed for 305 s.

From the integrated area at discharge condition in Supplementary Fig. S2 and the current, the stored energy can be calculated to be 0.0614 mWh. Therefore, the energy density based on the weight of the total weight of both electrodes (9.2 mg) can be calculated to be 6.7 Wh/kg.

In addition, the capacity of electrical charge can be calculated to be 32.6 mAh/g based on the weight of carbon material in single electrode (4.1 mg), the current (1.582 mA) and discharge time (305s).

3. Calculation details for energy density, capacity of electrical charge

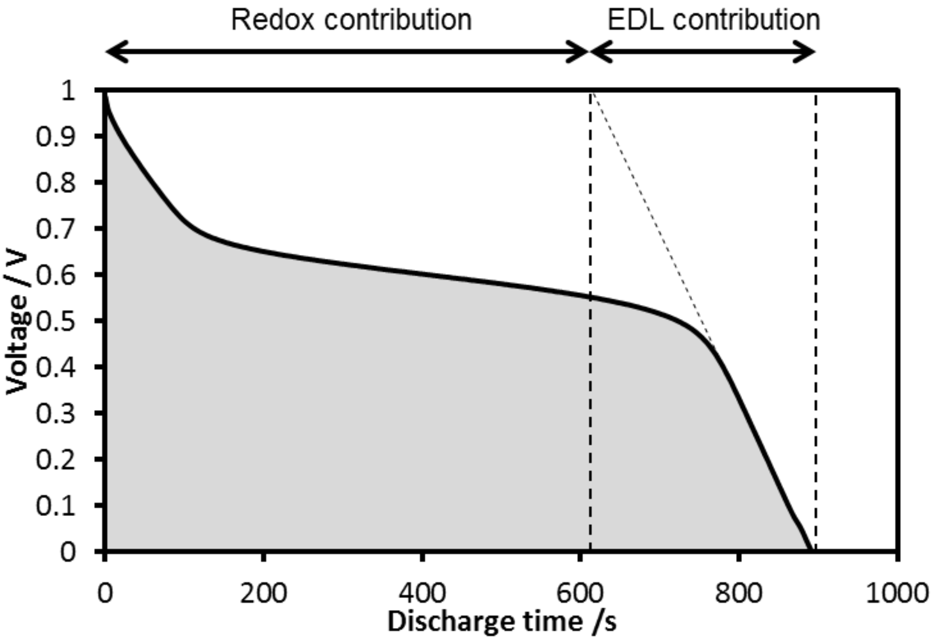

Supplementary Fig. S3 Discharge profile of the redox capacitor (TCHQ-AQ) in 0.5 M H_2SO_4 aqueous solution at 0.28 A/g.

The voltage profile of the redox capacitor (TCHQ-AQ) in discharge is shown in Supplementary Fig. S3. The total weight of two electrodes is 8 mg. The weight of carbon material and organic compound in one electrode is 2.51 mg and 1.07 mg, respectively. The charge and discharge current was fixed to be 1.152 mA. Therefore, the current density is 0.28 A/g based on the weight of single electrode (4 mg).

From the integrated area at discharge condition (grayish area) in Supplementary Fig. S3, the current (1.152 mA), the energy density based on the total weight of both electrodes (8 mg) can be calculated to be 20.3 Wh/kg.

On the other hand, the total capacity of electrical charge can be calculated to be 0.286 mAh, considering the current (1.152 mA) and total discharge time (894s). By extrapolating the galvanostatic discharge profile in the range from 0 to 0.4 V, the contributions of EDL and redox reaction can be estimated to be 0.0874 mAh and 0.199 mAh, respectively. Considering the weight of carbon material (2.51 mg) and organic compound (1.07 mg) in one electrode, the capacities of electrical charge derived from the EDL and the redox reactions can be estimated to be 34.8 mAh/g-carbon based on the carbon weight in single electrode and 186 mAh/g-(AQ or TCHQ) based on the organic compound weight in single electrode, respectively.

4. Structural analysis of organic compounds supported on nanoporous carbon

4.1 Support of low molecular weight organic compounds in the nanometer-scaled pores of carbon materials

To find a suitable carbon material, we tested three types with different pore structures. The less-porous carbon exhibited a much smaller Brunauer-Emmett-Teller (BET) surface area (110 m^2/g) and pore volume (0.37 cm^3/g). A carbon with pores of several tens of nanometers had a relatively larger BET surface area (1200 m^2/g) and the largest pore volume (3.14 cm^3/g) among the samples. The nanoporous activated carbon had the largest BET surface area (3070 m^2/g), although the pore volume (1.70 cm^3/g) was inferior to the carbon with pores of several tens of nanometers.
Supplementary Fig. S4 shows the pore size distributions of the carbon materials estimated using the Barrett-Joyner-Halenda (BJH) method, with pore radii ranging from ~1 to 100 nm. The vertical axis in the figure has the same dimension of volume per weight. In the BJH method, we assumed that the pores had cylindrical shapes. In the case of the carbon with pores of several tens of nanometers, the major pore size was several tens of nanometers, and the pore sizes were widely distributed over the entire range. In the case of the nanoporous carbon, the predominant pore size was less than 10 nm; pores larger than this were rare. By comparing these carbon materials with different pore size distributions, we could discuss the optimal pore size of a carbon support for organic compounds in a supercapacitor device.

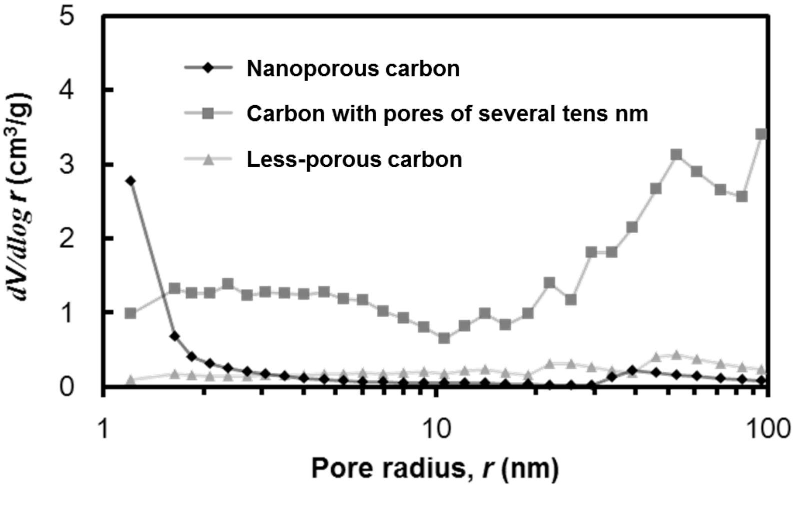

Supplementary Fig. S4 Pore size distribution for less-porous carbon, carbon with pores of several tens nanometers, and nanoporous carbon.

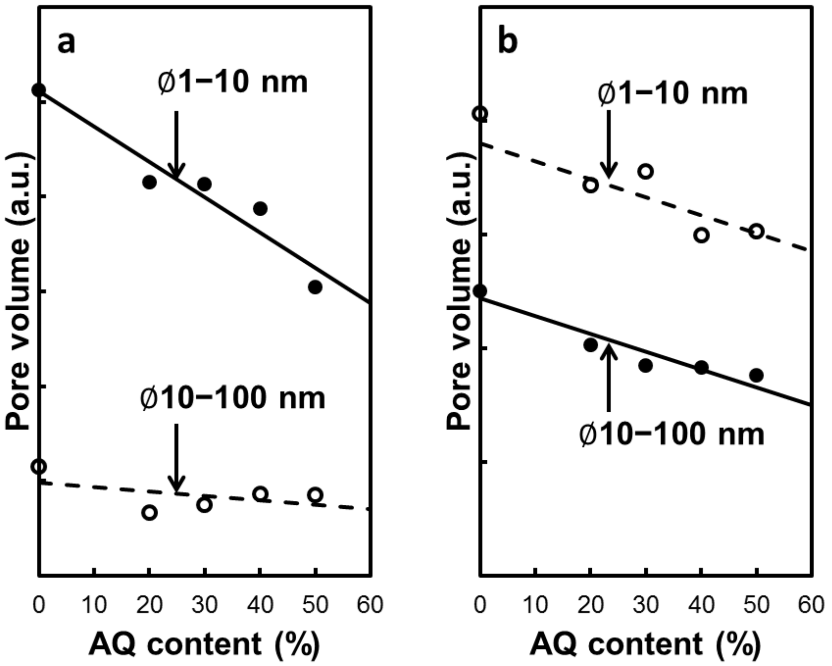

Supplementary Fig. S5 Pore volume for (**a**) nanoporous carbon and (**b**) carbon with pores of several tens nanometers as a function of AQ content in a carbon-AQ composite.

To determine the dominant pore size for the support of low molecular weight organic compounds in nanoporous carbon, we investigated the pore size distribution by varying the AQ loading. The preparation procedure was the same as that indicated in the methods section in the paper. Here, we focused on pores with diameters of less than 100 nm, and classified them into two groups, i.e., pores in the range 1–10 nm diameter and those in the range 10–100 nm diameter. In Supplementary Fig. S5, we compared the pore volume per weight of carbon materials in the two groups as a function of the AQ content. Assuming that the AQ itself doesn't have pore structure, the decrease in the pore volume resulted from the filling of the pores by AQ.

In the case of the nanoporous carbon with a dominant pore size of less than 10 nm diameter (Supplementary Fig. S5**a**), the decrease in the volume of the pores was significant. In the case of the carbon with pores of several tens nanometers (Supplementary Fig. S5**b**), the pore volumes for both sizes showed nearly the same decrease with increasing AQ content. These results indicated that, in both cases, pores of 1–10 nm diameter were filled by AQ, and especially in the case of nanoporous carbon, AQ was dominantly supported in the pores with diameters of less than 10 nm.

4.2 Structure of AQ in the nanometer-scaled pores of carbon materials

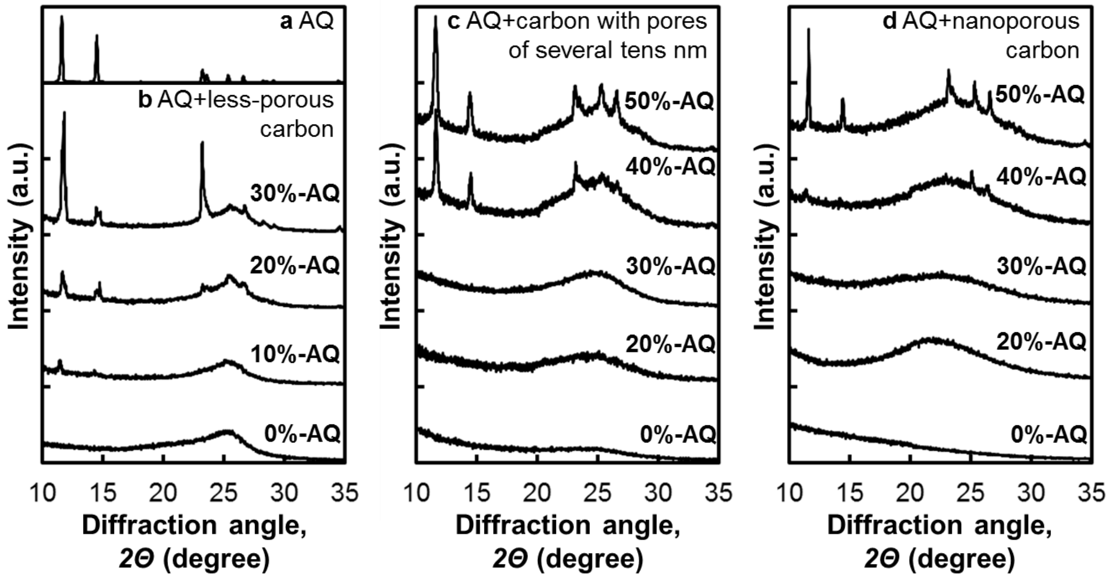

Supplementary Fig. S6 XRD patterns for (**a**) bulk AQ and carbon materials with various AQ contents, (**b**) less-porous carbon, (**c**) carbon with pores of several tens of nanometers, and (**d**) nanoporous carbon.

XRD patterns of AQ supported by carbon materials are shown in Supplementary Fig. S6. In the case of the less-porous carbon shown in Supplementary Fig. S6**b**, even at a small weight ratio of AQ to the carbon (1:9), the peaks corresponding to the crystalline AQ shown in Supplementary Fig. S6**a** were observed. This means that large particles of crystalline AQ are held in or on the less-porous carbon. The peaks were sharpened as the ratio of AQ to the carbon increased.

On the other hand, in the cases of the carbon with pores of several tens of nanometers and the nanoporous carbon in Supplementary Figs. S6**c** and **d**, respectively, until an AQ content of 30 wt% was achieved, the peaks corresponding to crystalline AQ were undetectable. These results suggested that, at the lower content, the organic compound was held in the nanoporous carbon with a less-crystalline or nanocrystalline structure. Above 40 wt%, the AQ peaks emerged, suggesting that larger particles of crystalline AQ were present.

Next, we investigated the organic compound fixed in the nanoporous carbon with less-crystalline or nanocrystalline structure by ^{1}H-NMR. Generally, NMR signals for solid samples are broadened compared to those from liquid samples. The broad nature of the peaks can be attributed to the restricted motion of the sample.

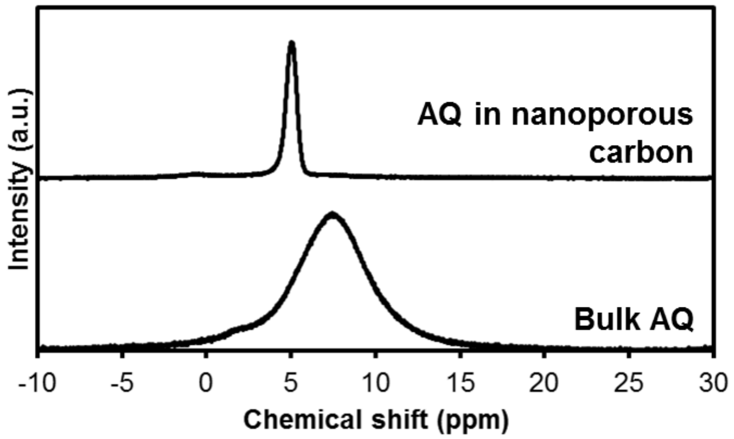

Supplementary Fig. S7 NMR spectra for AQ held in nanoporous carbon (upper) and bulk AQ (lower), obtained at a spin rate of 15 kHz at room temperature

Compared with bulk AQ showing a relatively broad signal, AQ held in nanoporous carbon yielded a sharp signal (Supplementary Fig. S7. This indicated that AQ in the nanometer-scale pore had a less-ordered structure, in which larger degrees of freedom, such as rotation and vibration, should be allowed for the AQ molecule compared with the bulk AQ. The XRD analysis, which suggested that the AQ held in the nanoporous carbon had a less-crystalline or nanocrystalline structure, was consistent with such NMR results. Moreover, the chemical shift of the AQ in the nanometer-scaled pore was observed at higher magnetic field. This shift can be assigned to the shielding effects of the graphene plane.[1] It is known that the ring current induced in the graphene plane results in the shift to lower frequency for the adsorbed molecule.

This suggests, therefore, that in the case of the AQ held in nanoporous carbon, the AQ which exists close to the carbon surface is dominant compared to the bulk AQ. Strong interactions between the graphene planes in the carbon materials and the aromatic rings in AQ should stabilize the absorbed organic compounds in the nanometer-scaled pores with less-crystalline or nanocrystalline structures.

4.3 Improvement of cycle performance by the support of organic compounds in the nanometer-scaled pores of carbon materials

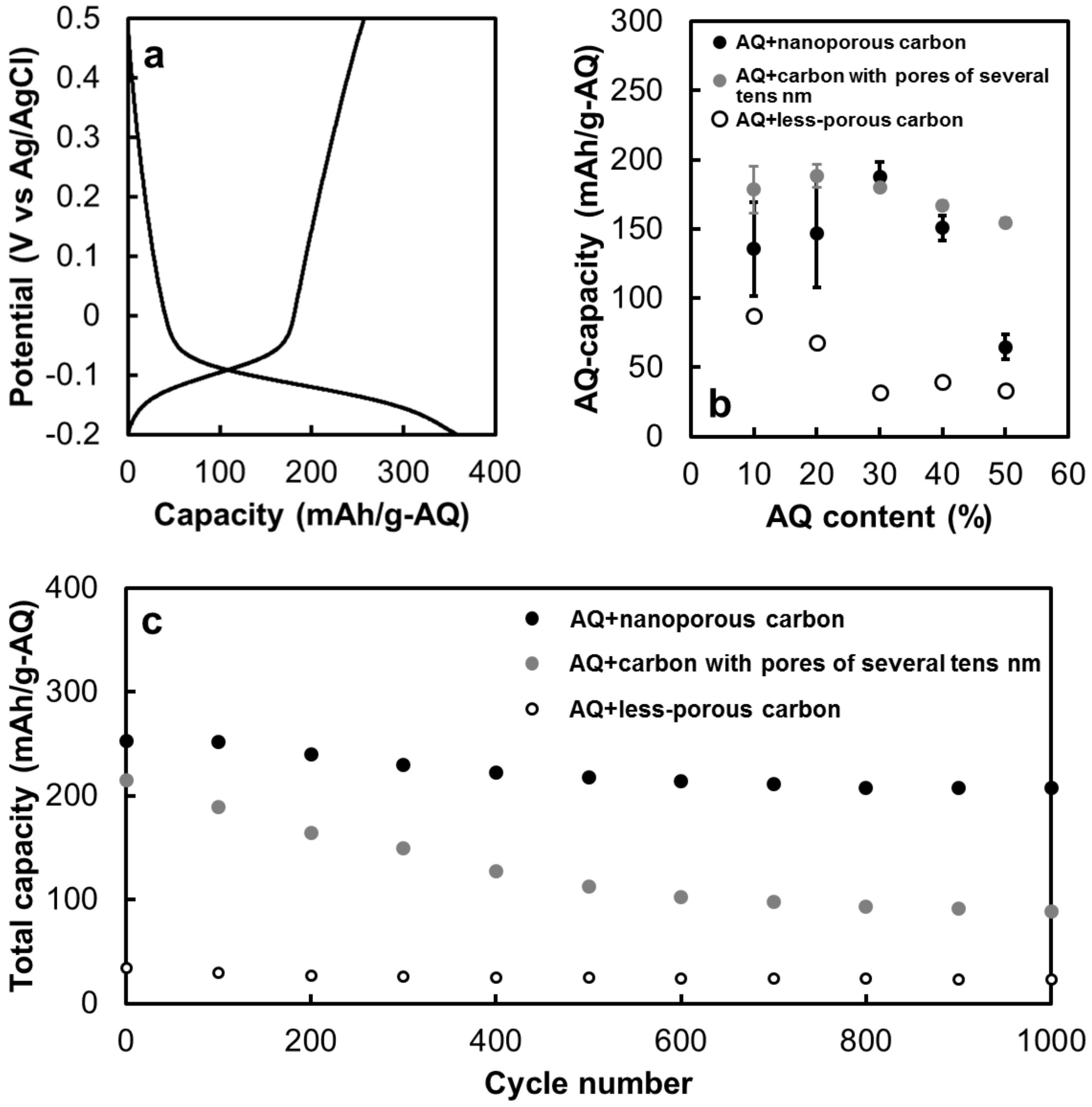

Supplementary Fig. S8 (**a**) Example of charge-discharge profiles for AQ held in nanoporous carbon. (**b**) Specific discharge capacities derived from AQ on the basis of the AQ amount as a function of AQ content. (**c**) Cycle performances of AQ supported by various carbon materials.

The electrochemical measurements were conducted using a three-electrode system indicated in the first section of the Supplementary Information to determine the electrochemical performance of the electrode consisting of organic compound.

Representative charge/discharge capacity profiles of the electrodes consisting of AQ

supported by nanoporous carbon are shown in Supplementary Fig. S8**a**. The electrical measurement was conducted as for single electrode characteristics. In the range from −0.15 to −0.05 V (vs. Ag/AgCl), the charge/discharge plateau resulting from the proton insertion/extraction reaction to/from AQ was observed. The electrical conductivity of the carbon materials facilitated the electrochemical reaction of the non-conductive AQ. The near-linear profile in the other range was derived from the EDL contribution of the carbon support. This EDL contribution to the total capacity becomes larger in the case of a lower load of organic compound.

Next, to determine the specific discharge capacity derived from only the organic compound, we estimated the EDL contribution by extrapolating the discharge capacity profile in the range from 0 to 0.5 V (vs. Ag/AgCl), where the AQ reaction does not take place, thus removing it from the total capacity. The specific discharge capacities derived from AQ as a function of the weight ratios of AQ to the sum of the carbon-AQ complexes are shown in Supplementary Fig. S8**b**. Because the discharge capacity profile resulting from EDL was not entirely linear with respect to the voltage, the error range was larger in the case of a lower load of organic compound, where the EDL contribution was larger.

In the case of the less-porous carbon, the specific discharge capacities derived from AQ were quite low over the whole range investigated. The specific capacitance became much lower as the AQ content increased. On the other hand, in the cases of the carbon with pores of several tens of nanometers and the nanoporous carbon, until the AQ content reached 30 wt%, the specific capacities retained large values; above 40 wt%, the specific capacities decreased as the AQ content increased. Interestingly, such low discharge capacities derived from AQ seem to correlate with the appearance of the crystalline AQ peaks in the XRD patterns shown in Supplementary Fig. S6.

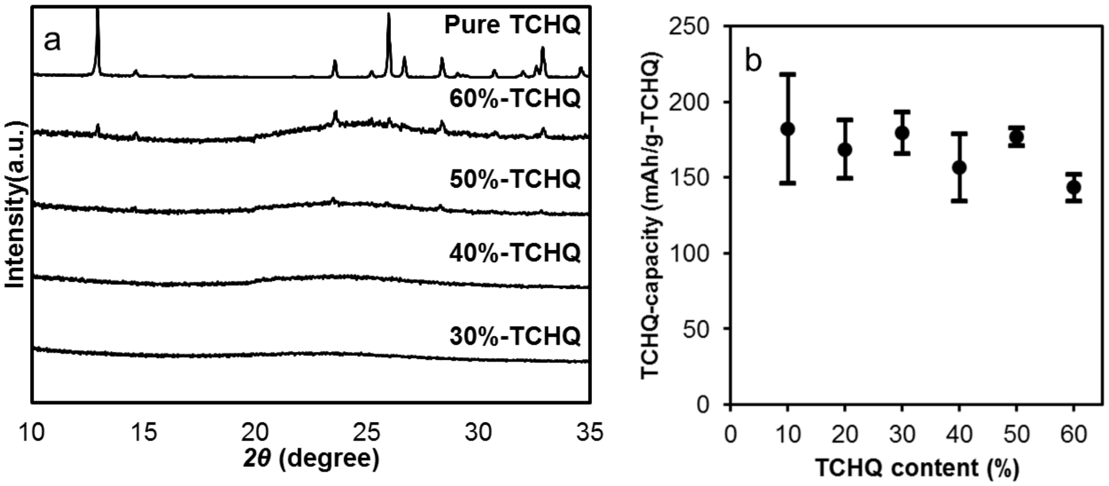

Supplementary Fig. S9 (**a**) XRD patterns for bulk TCHQ and nanoporous carbon with various TCHQ contents. (**b**) Specific discharge capacities derived from TCHQ on the basis of the TCHQ amount as a function of TCHQ content.

This behavior can be seen in the case of TCHQ. In Supplementary Fig. S9, we show XRD patterns for bulk TCHQ and nanoporous carbon with various TCHQ contents, and specific discharge capacities derived from TCHQ as a function of TCHQ content. In the cases of TCHQ, until the TCHQ content reached 50 wt%, the specific capacities retained large values; above 60 wt%, the specific capacities decreased as the TCHQ content increased. Such decrease in the utilization rate of TCHQ seems to correlate with the appearance of the crystalline TCHQ peaks in the XRD patterns.

These accordance indicates that the support of the organic compound in the nanoporous carbon with a less-crystalline or nanocrystalline structure is preferable to enhance the utilization rate of the organic compound in the supercapacitor.

Next, we investigated cycle performance. Supplementary Fig. S8**c** shows the cycle performance of the total capacities for various carbon materials with 30 wt% AQ. Compared to the results for the carbon with pores of several tens of nanometers, the nanoporous carbon showed superior cycle performance. After 1000 cycles, the retention of the capacity was above 80%. We believe that this difference is caused by the size difference of the pore holding the organic compound. It is known that the nanoporous carbon used in this study has abundant nanopores with complicated branching structures.[2] As mentioned in previous sections, for this particular carbon, the organic compound is held in nanometer-scaled pores, reducing the likelihood that the organic compound would leach from the carbon matrix. Moreover, in these pores, the organic compound has a less-crystalline/nanocrystalline structure due to its interactions with the

supporting carbon surface, such as hydrophobic and π-π interactions. Such interactions between the organic compound and carbon surface should be enhanced as the pore size decreases, and may help to prevent dissolution into solution.

From these results and discussion, the support of organic compounds in nanometer-scaled pores with less-crystalline/nanocrystalline structure was found to facilitate the high utilization rate of organic compounds with superior cycle performance.

5. Cycle performance for the redox capacitor (TCHQ-DCAQ)

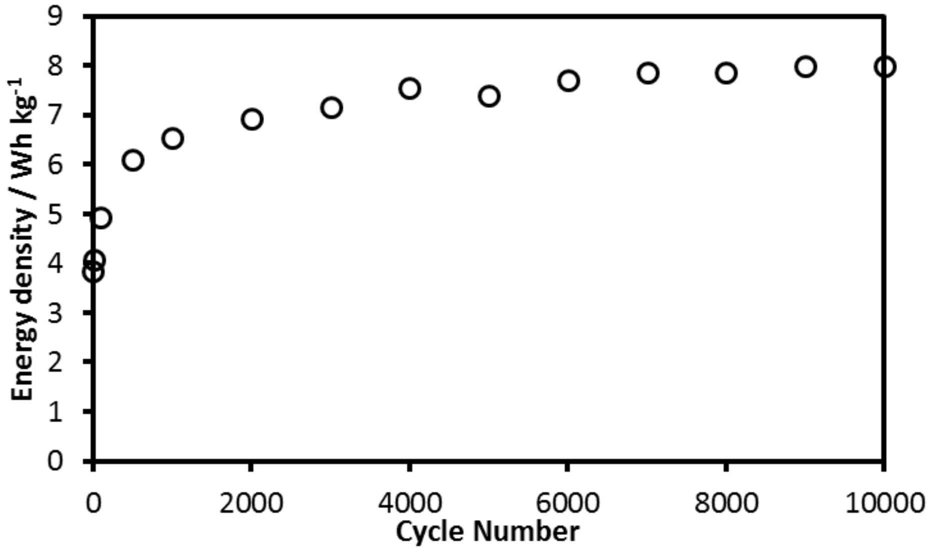

Supplementary Fig. S10 Rechargeable energy density of the redox capacitor (TCHQ-DCAQ) in 0.5 M H_2SO_4 aqueous solution at 2.6 A/g as a function of cycle time,

Supplementary Fig. S10 shows the rechargeable energy density of the redox capacitor using the DCAQ and TCHQ couple as a function of cycle time. Similar to the redox capacitor that used the AQ and TCHQ couple (~20 Wh/kg at 0.28 A/g and ~12 Wh/kg at 2.8 A/g), the energy density decreased as increasing the current density (~15 Wh/kg at 0.26 A/g and ~8 Wh/kg at 2.6 A/g).

From this experiment for the cycle performance of the redox capacitor (TCHQ-DCAQ), the degradation of rechargeable energy density was not observable after 10000 cycles.

6. Selection of organic compounds as proton acceptor and proton donor

In the case of a redox capacitor that employs a proton rocking-chair system between the organic compounds in the couple, a large potential difference between the proton-acceptor and proton-donor organic materials is preferable. Therefore, the quinones which showed lower reaction potentials than that of benzoquinone (BQ) (or hydroquinone, (HQ)) were regarded as candidates for proton acceptors. HQ and hydroquinonic compounds which showed higher reaction potentials than HQ were regarded as proton-donor candidates.

Supplementary Table S1. Quinones and hydroquinones investigated as proton acceptors and proton donors

Proton acceptor

	2,5-dihydroxy-*p*-benzoquinone (DHBQ)	Naphthoquinone (NQ)	2,5-dimethoxy-*p*-benzoquinone (DMBQ)	Anthraquinone (AQ)
**Structure**	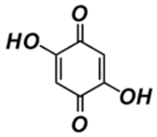	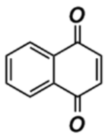	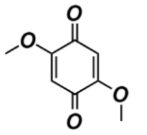	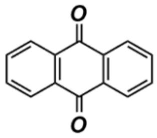
**Molecular weight (g/mol)**	140	158	168	208
**Ideal capacity (mAh/g)**	383	339	319	257
**Redox potential (V vs. Ag/AgCl)**	0.15	0.21	0.19	−0.16

Proton donor

	Hydroquinone (HQ)	Tetrachloro-hydroquinone (TCHQ)
**Structure**	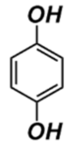	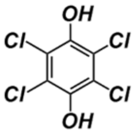
**Molecular weight (g/mol)**	110	248
**Ideal capacity (mAh/g)**	487	216
**Redox potential (V vs. Ag/AgCl)**	0.47	0.50

The quinones and hydroquinones investigated as proton acceptors and donors in this study are listed in Supplementary Table S1, respectively. Their molecular weights and ideal capacities in two-electron reactions are also shown. Nanoporous carbon was employed as the support for the organic compounds, and the electrodes consisted of the organic compound (27 wt%), nanoporous carbon (63 wt%), and PTFE (10 wt%).

The redox potentials of the proton insertion/extraction reactions calculated as the averaged potentials of the anodic and cathodic peak potentials in the voltammograms are also listed in Table S1. As is well known, adding electron-donating groups, such as hydroxyl or methoxyl groups, or increasing the number of aromatic rings is effective for increasing the reaction potential, and adding electron-accepting groups, such as halogens, is effective for decreasing the reaction potential.

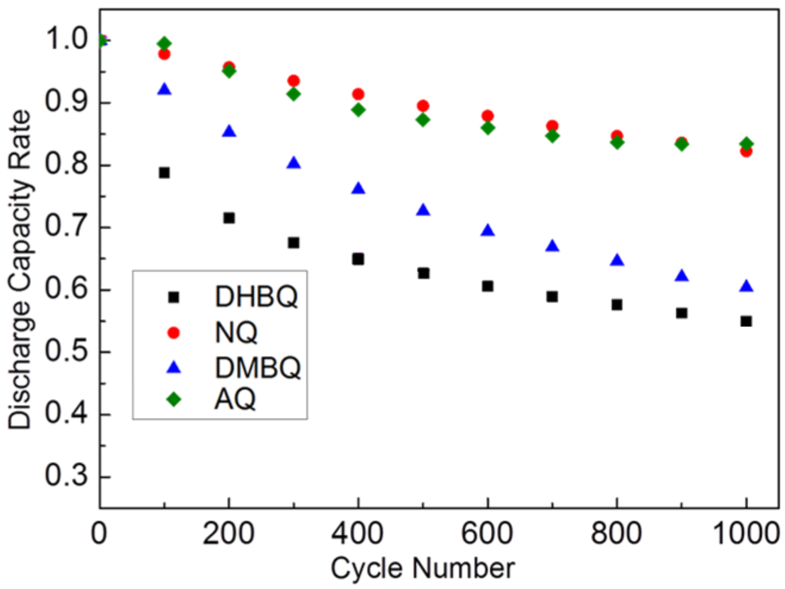

Supplementary Fig. S11 Retention rates of discharge capacities for various quinones as proton acceptors at 10 C rate for the ideal capacity of quinonic compounds.

The retention rates of the discharge capacities for the various quinones as proton acceptors are shown in Supplementary Fig. S11. Among them, DHBQ showed the worst cycle performance. The addition of hydroxyl groups, which is hydrophilic group, should promote dissolution into the aqueous solution. On the other hand, the retention rate of DMBQ having hydrophobic group, methoxy group, was higher than that of DHBQ. Moreover, AQ and NQ showed superior cycle performance within the group. It is likely that the increment in the number of aromatic rings, the enhancement of hydrophobic

interactions, and the π-π interactions between the supporting carbon surface and the organic compound contributed to the prevention of dissolution. This trend corresponded to a previous report[3] in which an increasing number of aromatic rings was effective in preventing dissolution and enhancing cycle performance.

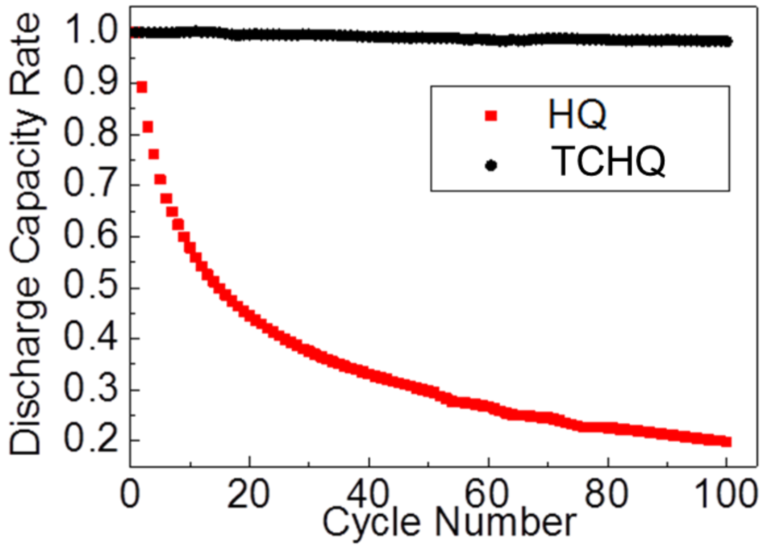

Supplementary Fig. S12 Retention rates of discharge capacities for various hydroquinones as proton donors at 2 C rate for the ideal capacity of quinonic compounds.

The retention rates of the discharge capacities for HQ and TCHQ as proton donors are shown in Supplementary Fig. S12. HQ showed a quite low retention rate. In contrast, TCHQ showed better cycle performance, and the retention rate after 100 cycles was above 95%. Most likely, the low solubility of TCHQ in aqueous solution due to its hydrophobic chloro substituents contributed to the high retention rate.

In short, among the compounds investigated in this study, we concluded that a couple comprising the organic compounds AQ and TCHQ would be preferable for the fabrication of a proton rocking-chair capacitor with good cycle performance and a larger potential difference.

REFERENCES

1. Harris, R. K., *et al*. A magic-angle spinning NMR study into the adsorption of deuterated water by activated carbon. *Carbon* **34**, 1275–1279 (1996).
2. Xing, W., *et al*. Superior electric double layer capacitors using ordered mesoporous carbons. *Carbon* **44**, 216–224 (2006).
3. Yao, M., Yamazaki, S., Senoh, H., Sakai, T. & Kiyobayashi, T. Crystalline polycyclic quinone derivatives as organic positive-electrode materials for use in rechargeable lithium batteries. *Mater. Sci. Eng. B* **177**, 483–487 (2012).
